# Risk factors of low back pain and the relationship with sagittal vertebral alignment in Tanzania

**DOI:** 10.1186/s12891-019-2953-x

**Published:** 2019-12-04

**Authors:** Masataka Tatsumi, Egfrid Michael Mkoba, Yusuke Suzuki, Yuu Kajiwara, Hala Zeidan, Keiko Harada, Tsubasa Bitoh, Yuichi Nishida, Kengo Nakai, Kanako Shimoura, Tomoki Aoyama

**Affiliations:** 1 Present address: Department of Physical Therapy, Human Health Sciences, Graduate School of Medicine, Kyoto University (Japan), 53 Kawara-cho, Shogoin, Sakyo-ku, Kyoto, 606-8507 Japan; 2 Present address: School of Physiotherapy, Kilimanjaro Christian Medical Centre, Moshi (United Republic of Tanzania), P.O.Box 2202, Moshi, Tanzania

**Keywords:** Low back pain (LBP), Sub-Sahara Africa (SSA), Tanzania, Pelvic tilt, Lumbar lordosis, Thoracic kyphosis

## Abstract

**Background:**

LBP is a common and serious problem affecting vast populations of the world. However, only few studies on LBP in sub-Saharan Africa have been conducted. Studies report that LBP and pelvic angle are interrelated, and African residents have a high pelvic tilt. The strategy to prevent LBP should focus on activities that promote holistic health. For that purpose, it is important to grasp the state of LBP and how it affects people’s lifestyle in Tanzania to clarify the direction of implementation of physiotherapy treatment and reduce the incidences of LBP among adults. This study aimed to investigate the prevalence and presentation of low back pain (LBP) and the relationship between anthropometric measurements and LBP among people in Moshi city, Kilimanjaro region Tanzania.

**Methods:**

Following signing consent forms, participants were given questionnaires regarding LBP and then grouped accordingly into either asymptomatic or symptomatic cohorts. Anthropometric measurements of participants’ height, weight, curvature of the spine, and pelvic angle were obtained.

**Results:**

A Mann-Whitney U test analysis showed a significant difference in pelvic angle, body mass index (BMI), and thoracic kyphosis angle between the asymptomatic group and the symptomatic group. No significant differences in lumbar lordosis angle or abdominal muscle strength were found between the two groups.

**Conclusions:**

A person with symptomatic LBP in Tanzania has a large anteversion of the pelvic tilt and a thoracic kyphotic posture. This study shows a relationship between sagittal spinal alignment and LBP in Tanzania, which could allow for prospective identification of subjects prone to developing LBP in the future.

## Introduction

Low back pain (LBP) is a common and serious health problem affecting the vast majority of the world’s population [1]. It is the most prevalent musculoskeletal condition in developed countries and is widely recognized as the leading cause of disability in high-income countries (HICs) and developed countries [2]. However, in low-income countries, particularly sub-Saharan Africa (SSA), very little is known about LBP [3]. The prevalence of LBP in adults in Africa was assumed to be lower than that in developed countries; however, a previous study showed that its prevalence is 32% and rising [3], suggesting that further research on strategies for the prevention and management of LBP in Africa is necessary. Although studies focusing on African countries have been conducted recently, most of the research has been performed in the Republic of South Africa and Nigeria. In addition, SSA tends to be grouped collectively, but the region is huge and there are differences between the constituent countries [4].

It is imperative, therefore, that more research is conducted on strategies for the prevention and management of LBP in Africa. The epidemiology of LBP has been well-described, but most studies are limited to HICs, and investigation of the factors related to LBP focusing on adults in Africa, including Tanzania, is lacking [5]. Despite the high prevalence (73.6%) of LBP among nurses in this country [6]. studies investigating the occurrence of LBP within Tanzania are very limited. Furthermore, there are reports in HICs that LBP and pelvic angle are interrelated [7], and Afro-African are generally reported to have a high pelvic tilt [8].

Since LBP interferes with not only work but also activities such as exercise and sleeping, strategies to prevent LBP contribute for promoting holistic health as physiotherapy. Therefore, it is important to understand the condition of LBP and how it affects people’s lifestyle in Tanzania in order to guide treatments, such as physiotherapy, and reduce the incidence of LBP among adults. This study aimed to investigate the prevalence and presentation of LBP, and the relationship between anthropometric measurements and LBP among people in Tanzania. In this case, we investigated Moshi city, Kilimanjaro region Tanzania.

## Methods

The study was conducted in the school and the department of physiotherapy at Kilimanjaro Christian Medical Centre (KCMC). Written informed consent was obtained from all participants. Sixty-eight subjects participated, each completing questionnaires regarding LBP. Physical anthropometric measurements on each subject were also performed. Height was measured using a Height Meter HM 200P (AS One Corporation, Japan). An Omron weighing scale model HBF-214-EBW (Omron Healthcare Co. Ltd., Kyoto, 617–0002 Japan) was used to measure weight. Pelvic angle was measured using a PALM palpation meter (Performance Attainment Associates, St. Paul, MN, USA), a pelvic-leveling device that combines the features of a measurement caliper and an inclinometer. The PALM palpation meter” has been validated in several previous studies [9–12]. The subjects stood with legs aligned with their shoulders, positioned their arms by the side of their body and looked at a fixed point ahead to control for posture sway. The anterior superior iliac spines (ASIS) and posterior superior iliac spines (PSIS) were palpated and the caliper tips of the PALM was positioned against the lower end of the ASIS and PSIS. A positive angle value meant anterior pelvic rotation, while negative angle value meant posterior pelvic rotation. Pelvic alignment data was measured two times and their average was used for analyses. Spinal Mouse® (Idiag, Fehraltdorf, Switzerland), a noninvasive device that is combined with a computer program was used to measure sagittal thoracic and lumbar curvatures. The subjects’ spines were traced using spinal mouse from C7 to S3. The angle of LL was the angle from Th12/L1 to L5/S1 and these angles were calculated automatically. The Spinal Mouse has also been validated in a previous study [13–15]. A positive angle value meant posterior spinal curvature, while negative angle value meant anterior spinal curvature. Spinal alignment was measured 3 times and their average was used for analyses. In addition, subjects’ truncal function was evaluated by the Kraus–Weber test. The Kraus-Weber test assesses the strength of core muscles with simple actions [16].

In this study, we defined LBP as the pain localized below the costal margin and the inferior gluteal folds without sciatica, the score of NRS (Numerical Rating Scale) is 4 or more and chronic when it persists for 12 weeks. We conducted the survey focusing on physical characteristics as predictors of LBP.

Based on questionnaire results, subjects were categorized into two groups: asymptomatic (*n* = 52; age, 20–45 years) and symptomatic individuals (*n* = 16; age, 20–55 years).

Data was then analyzed to investigate any correlation of LBP and pelvic angle, and compare differences in pelvic angle, spinal curvature, and trunk muscle strength between the asymptomatic and symptomatic groups. Differences in continuous variables were assessed using an independent t-test or Mann–Whitney U test. All statistical analyses were carried out using SPSS version 20.0 (IBM Corp., Armonk, NY, USA), and a *p*-value < 0.05 was considered as statistically significant.

## Result

There are 16 subjects on symptomatic groups for 68 subjects, which accounts for about 20% of the total on this study. Table 1 shows the physical characteristics of the participants in each group, presenting the mean values, standard deviations, and ranges for age, height, weight, and body mass index (BMI) of the participants in each group. Table 2 compares thoracic kyphosis angle, lumbar lordosis angle, abdominal muscle strength, and pelvic angle between the asymptomatic and symptomatic groups. According to the Mann–Whitney U test, a significant difference was observed in pelvic angle, BMI, and thoracic kyphosis angle between the asymptomatic and the symptomatic groups. No significant difference was observed between the lumbar lordosis angle and the abdominal muscle strength between the 2 groups.

**Table 1:** Physical characteristics of participants

	Asymptomatic group (*n* = 52)	Symptomatic group (*n* = 16)	*p* value
Age (years)	23 (10.3)	31 (18.5)	0.04*
Height (cm)	167.7 (11.5)	166.7 (10.2)	0.62
Weight (kg)	61 (13.0)	75 (21.4)	0.02*
Body mass index (kg/m^2^)	22.2 (3.7)	26.2 (4.4)	0.00**
median (IQR)

**Table 2:** Parameters of sagittal spinal, pelvic alignment, and abdominal muscle strength in asymptomatic and symptomatic groups

	Asymptomatic group (*n* = 52)	Symptomatic group (*n* = 16)	*p* value
Pelvic angle -Rt (°)	7 (4)	12 (4)	0.00**
Pelvic angle -Lt (°)	7 (3.3)	10.5 (6.3)	0.00**
Lumbar lordosis(°)	30.5 (14.6)	32.2 (13.9)	0.29
Thoracic kyphosis(°)	44 (13.3)	51.3 (8.2)	0.02*
Abdominal muscle strength	8 (8.3)	6.5 (6.75)	0.81
median ± (IQR)			

*P* values from mean comparisons are also reported

Statistically significant correlation coefficient *(*P* < 0.05) or **(*P* < 0.01)

## Discussion

Several agreements on the classifications of LBP have been documented. These classifications fall into three major domains described as 1) physical, 2) psychological, and 3) pathological. Several studies have reported the predictors of LBP, of which we site physical stress, [17], psychological stress [18, 19], personal characteristics [20],and physical characteristics [21] as predictors of LBP. These factors surround primarily ergonomic conditions in which body mechanics and extrinsic factors including work conditions play an important role in developing the pain.

Postural abnormalities may play a role in the occurrence of LBP by creating concentrations of stress. Nevertheless, this assumption remains speculative because of the absence of criteria for normal posture. Several studies have investigated sagittal spinal alignment and pelvic angle in lumbar disorders [22, 23]. In two studies evaluating sagittal lumbar alignment and LBP, no differences were found in the parameters of the lumbar or pelvic alignment in subjects with low back pain compared with normal individuals [24, 25]. In contrast, some individuals exhibited an increased lumbar lordosis compared with those without LBP [26]. The influence of sagittal spinal and pelvic alignment on LBP needs further elucidation.

Additionally, despite considerable study of the epidemiology of LBP, little is known about the factors related to LBP in adults in Tanzania [5], as studies in this population are lacking. In the present study, the results contribute to a better understanding of the relationship between sagittal alignment and LBP. This could potentially promote identification of subjects prone to develop LBP in Tanzania in the future based on their sagittal spinal and pelvic alignment.

This study found significant differences in pelvic angle and degree of thoracic segmental kyphosis between subjects with and without LBP. From this, we infer that a person with LBP symptoms in Tanzania has a larger anteversion of the pelvic angle and has a postural thoracic kyphosis. In contrast, no significant difference was observed in degree of lumbar lordosis between asymptomatic and symptomatic subjects. Therefore, we infer that large lumbar lordosis in Tanzania is not directly linked to the cause of LBP (Table 2).

The impact of sagittal vertebral alignment on the treatment of spinal disorders is of critical importance. The principles of sagittal balance are vital to achieve optimum outcomes when treating spinal disorders, since a failure to recognize malalignment in this plane can have significant consequences for the patient in terms of pain and deformity [27]. Normally, the spine is known to have a lordotic curves in the cervical and lumbar regions and a kyphotic curve in the thoracic region. A position correlation is found between thoracis kyphosis and lumbar lordosis. The degrees and shape of these curvatures allow an equal distribution of forces across the spinal column [28]. Pain and deformity result by imbalance and loss of equilibrium between these structures by pathological processes. A malalignment in the sagittal plane is presented as an exaggeration or deficiency of normal lordosis or kyphosis. This malalignment is usually accompanied by pain and functional disability [26, 29]. As a result, the pelvic and lower limb posture compensate for the imbalance to restore normal alignment.

A prior study [30] supports the concept that the pelvis and spine of asymptomatic adults can be considered in the sagittal plane as an open linear chain linking the head to the pelvis. Moreover, this concept implies that a change in shape or orientation at any level will effect a change on adjacent segments and will modify their shape. Therefore, several reports recommend muscle strength training of core muscles including the abdominal muscles aiming to improve hyperlordosis of the lumbar segment to prevent LBP [31–33]. Considering the results of the present study, this approach to improve trunk muscle strength is not enough to ameliorate LBP. Anterior and posterior pelvic muscles cause a disrupted pelvic angle when they are imbalanced. This imbalance is expressed by an anterior tilt of the pelvis by the quadriceps muscles and iliopsoas muscles, while the posterior tilt is caused by the hamstrings muscle pulling posteriorly [34]. Accordingly, for those who have LBP with lumbar hyperlordosis, correcting muscle flexibility and strength on abdominal and hip joint is necessary to improve anterior pelvic tilt [35, 36].

Our results suggest that excessive thoracic kyphosis is also associated with LBP in the residents of Moshi city, Kilimanjaro, Tanzania. Thoracic kyphosis contributes to positioning the center of gravity of the body behind. It is thought that when there is a thoracic kyphosis, the burden increases on the waist as a motion strategy is taken to ensure postural stability [37]. Therefore, evaluation such as the flexibility and stability that contribute to kyphosis of the thoracic vertebrae are indispensable for exploring the causes of people with LBP in Tanzania.

In addition, the results suggest that obesity seems to be one of the main reasons why the hyperlordosis of the lumbar vertebrae did not show a significant difference from LBP in this study. BMI was classified according to the World Health Organization as underweight (BMI < 18.5), normal weight (BMI = 18.5–24.9), overweight (BMI = 25–29.9), obesity I (BMI = 30–34.9) and obesity II (BMI = 34.9–39.9). From the BMI results in Table 1, the group with LBP has a significantly higher obesity rate. Additionally, previous studies have shown that overnutrition is increasing in some Sub-Saharan African societies, particularly in urbanized areas with western lifestyles [38]. Obesity causes tissue thickening. Therefore, due to the thick soft tissue, it could be considered that the Spinal Mouse used to measure along the body surface could not accurately capture the true lumbar vertebral curves in more obese subjects. In region where there is no equipment used for diagnostic imaging, it is very important to understand the features of the morphology of the body to give a treatment program. In this case, to replace the diagnostic images, it is appropriate to use indices of the thoracic vertebrae and pelvis with less influence of soft tissue in the anthropometric measurement, such as the methods we have used.

A majority of the current international community consists of developing countries with a lower standard of health service than that of developed countries. In developing regions like Tanzania, where diagnostic imaging and enough research are lacking, it is important to understand the morphological features to reduce the risk of many local populations’ musculoskeletal problems, to give an efficient treatment program and to promote the concept of prevention. As this study focuses on LBP in one of the areas in Africa where little research on the subject has been performed, our results will contribute to reducing the risk of many local populations’ musculoskeletal problems and promote the concept of prevention. Aside from improving the quality of health service, this research can bring a sizeable beneficial impact on larger populations.

However, this study is a cross-sectional study, the causes and results are unclear. And in this study, the alignment of the pelvis and lumbar spine is measured only in the sagittal plane. The alignment of the lumbar spine where no significant difference was seen in the sagittal plane may have been caused by rotation in the horizontal plane, scoliosis in the frontal plane, etc. Furthermore, backache has also been reported to be related to age, mental aspect such as depression, smoking history, etc. These measurements are also essential for a more detailed evaluation.

## Conclusion

We investigated whether trunk strength, curvature of the thoracic and lumbar vertebrae, and inclination of the pelvis are related to low back pain in our study population of Tanzania residents. The results indicate that a person with LBP symptoms in Tanzania has a large anteversion of the pelvic angle and a posture of thoracic kyphosis, and demonstrate that evaluation should not only focus on hyperlordosis of the lumbar spine but also, and perhaps more importantly, on the anterior tilt of the pelvis and the mobility of the thoracic vertebrae with a large kyphosis. Overall, the results contribute to a better understanding of the relationship between sagittal alignment and LBP in Tanzania. Although much still remains to be understood, we believe that the results presented in this study significantly contributes to the existing literature.

## Data Availability

The datasets used and/or analysed during the current study are available from the corresponding author on reasonable request.

## References

[1] Hoy D (2014). The global burden of low back pain: estimates from the global burden of disease 2010 study. Ann Rheum Dis.

[2] Woolf AD, Pfleger B (2003). Burden of major musculoskeletal conditions. Bull World Health Organ.

[3] Louw QA, Morris LD, Grimmer-Somers K (2007). The prevalence of low back pain in Africa: a systematic review. BMC Musculoskelet Disord.

[4] Tabutin Dominique SB (2004). The demography of sub-Saharan Africa from the 1950s to the 2000s. Population.

[5] Volinn E (1997). The epidemiology of low back pain in the rest of the world*. A review of surveys in low- and middle-income countries*. Spine (Phila Pa 1976).

[6] Mwilila, M.C., Work-related low back pain among clinical nurses in Tanzania, in Dept. of Physiotherapy. 2010, University of the Western Cape: University of the Western Cape. P. None

[7] Chun SW (2017). The relationships between low back pain and lumbar lordosis: a systematic review and meta-analysis. Spine J.

[8] Mosner EA (1989). A comparison of actual and apparent lumbar lordosis in black and white adult females. Spine (Phila Pa 1976).

[9] Petrone MR (2003). The accuracy of the palpation meter (PALM) for measuring pelvic crest height difference and leg length discrepancy. J Orthop Sports Phys Ther.

[10] Azevedo DC (2014). Reliability of sagittal pelvic position assessments in standing, sitting and during hip flexion using palpation meter. J Bodyw Mov Ther.

[11] Herrington L (2011). Assessment of the degree of pelvic tilt within a normal asymptomatic population. Man Ther.

[12] Hagins Marshall, Brown Martha, Cook Clare, Gstalder Karen, Kam Michael, Kominer Gene, Strimbeck Katesel (1998). Intratester and Intertester Reliability of the Palpation Meter (PALM) in Measuring Pelvic Position. Journal of Manual & Manipulative Therapy.

[13] Barrett E, McCreesh K, Lewis J (2014). Reliability and validity of non-radiographic methods of thoracic kyphosis measurement: a systematic review. Man Ther.

[14] Livanelioglu A (2016). The validity and reliability of "spinal mouse" assessment of spinal curvatures in the frontal plane in pediatric adolescent idiopathic thoraco-lumbar curves. Eur Spine J.

[15] Kellis E (2008). Reliability of spinal range of motion in healthy boys using a skin-surface device. J Manip Physiol Ther.

[16] Kiss G (2019). Efficiency examination of a 6-month trunk prevention program among recruitment kayak-canoe athletes: a randomized control trial. J Back Musculoskelet Rehabil.

[17] Bigos SJ (1992). A longitudinal, prospective study of industrial back injury reporting. Clin Orthop Relat Res.

[18] Houtman IL (1994). Psychosocial stressors at work and musculoskeletal problems. Scand J Work Environ Health.

[19] Bongers PM (1993). Psychosocial factors at work and musculoskeletal disease. Scand J Work Environ Health.

[20] Deyo RA, Bass JE (1989). Lifestyle and low-back pain. The influence of smoking and obesity. Spine (Phila Pa 1976).

[21] Dempsey PG, Burdorf A, Webster BS (1997). The influence of personal variables on work-related low-back disorders and implications for future research. J Occup Environ Med.

[22] Evcik D, Yücel A (2003). Lumbar lordosis in acute and chronic low back pain patients. Rheumatol Int.

[23] Gautier J, Morillon P, Marcelli C (1999). Does spinal morphology influence the occurrence of low back pain? A retrospective clinical, anthropometric, and radiological study. Rev Rhum Engl Ed.

[24] During J (1985). Toward standards for posture*.* Postural characteristics of the lower back system in normal and pathologic conditions. Spine (Phila Pa 1976).

[25] Laird RA, Kent P, Keating JL (2016). How consistent are lordosis, range of movement and lumbo-pelvic rhythm in people with and without back pain?. BMC Musculoskelet Disord.

[26] Christie HJ, Kumar S, Warren SA (1995). Postural aberrations in low back pain. Arch Phys Med Rehabil.

[27] Takemoto M (2016). Sagittal malalignment has a significant association with postoperative leg pain in adult spinal deformity patients. Eur Spine J.

[28] Roussouly P, Nnadi C (2010). Sagittal plane deformity: an overview of interpretation and management. Eur Spine J.

[29] Hertel J, Dorfman JH, Braham RA (2004). Lower extremity malalignments and anterior cruciate ligament injury history. J Sports Sci Med.

[30] Berthonnaud E (2005). Analysis of the sagittal balance of the spine and pelvis using shape and orientation parameters. J Spinal Disord Tech.

[31] Kim JH (2013). The effect of the neurac sling exercise on postural balance adjustment and muscular response patterns in chronic low back pain patients. J Phys Ther Sci.

[32] Yoo WG (2017). Effect of modified leg-raising exercise on the pain and pelvic angle of a patient with back pain and excessive lordosis. J Phys Ther Sci.

[33] Carpenter DM, Nelson BW (1999). Low back strengthening for the prevention and treatment of low back pain. Med Sci Sports Exerc.

[34] Frigo C, Pavan EE, Brunner R (2010). A dynamic model of quadriceps and hamstrings function. Gait Posture.

[35] López-Miñarro PA (2012). Acute effects of hamstring stretching on sagittal spinal curvatures and pelvic tilt. J Hum Kinet.

[36] Workman JC (2008). Influence of pelvis position on the activation of abdominal and hip flexor muscles. J Strength Cond Res.

[37] Briggs AM (2007). Thoracic kyphosis affects spinal loads and trunk muscle force. Phys Ther.

[38] Ojo G. The Relationship between Skinfold Thickness and Body Mass Index in Estimating Body Fat Percentage on Bowen University Students. In: Adetola O, editor. Department of Anatomy, Faculty of Basic Medical and Health Sciences, Iwo, Osun State, Nigeria. IBBJ: International Biological & Biomedical journal; 2017. p. 138–144.

